# Balancing the influenza neuraminidase and hemagglutinin responses by exchanging the vaccine virus backbone

**DOI:** 10.1371/journal.ppat.1009171

**Published:** 2021-04-19

**Authors:** Jin Gao, Hongquan Wan, Xing Li, Mira Rakic Martinez, Laura Klenow, Yamei Gao, Zhiping Ye, Robert Daniels

**Affiliations:** Division of Viral Products, Center for Biologics Evaluation and Research, Food and Drug Administration, Silver Spring, Maryland, United States of America; Thomas Jefferson University, UNITED STATES

## Abstract

Virions are a common antigen source for many viral vaccines. One limitation to using virions is that the antigen abundance is determined by the content of each protein in the virus. This caveat especially applies to viral-based influenza vaccines where the low abundance of the neuraminidase (NA) surface antigen remains a bottleneck for improving the NA antibody response. Our systematic analysis using recent H1N1 vaccine antigens demonstrates that the NA to hemagglutinin (HA) ratio in virions can be improved by exchanging the viral backbone internal genes, especially the segment encoding the polymerase PB1 subunit. The purified inactivated virions with higher NA content show a more spherical morphology, a shift in the balance between the HA receptor binding and NA receptor release functions, and induce a better NA inhibitory antibody response in mice. These results indicate that influenza viruses support a range of ratios for a given NA and HA pair which can be used to produce viral-based influenza vaccines with higher NA content that can elicit more balanced neutralizing antibody responses to NA and HA.

## Author summary

Influenza vaccines are produced on a large scale to meet the annual U.S. and global demand. To efficiently produce the required number of influenza vaccine doses, virions are commonly used as the antigen source due to their high viral protein content. A draw-back to using virions is that the final antigen composition of the vaccine is determined by the inherent properties of the vaccine virus. While this approach for influenza vaccines is beneficial for the more abundant HA antigen, it likely limits the protective response generated by the less abundant NA antigen. Our results demonstrate that the NA and HA content in vaccine viruses can be optimized by changing the internal genes of the vaccine virus, thereby preserving the surface antigens. The increase in the virion NA content that was achieved elicited higher NA antibody titres and generated more balanced neutralizing antibody responses to HA and NA. Since HA and NA neutralizing antibodies are both protective, this approach could help to improve the suboptimal efficacy of current influenza vaccines and to generate vaccines that provide broader coverage against circulating strains.

## Introduction

Virions consist of a viral genome surrounded by an outer protein shell, or a viral protein-containing envelope (membrane). The main function of the viral surface proteins is to initiate the infection process by mediating specific interactions with the host cell, which is crucial for viral genome delivery. These essential functions and the accessibility of the surface proteins are some of the primary reasons many of them are also effective viral vaccine antigens. Accordingly, virions, which possess high surface protein content and are relatively easy to isolate, are commonly used as an antigen source for many vaccines including those against polio, hepatitis A and influenza viruses [1]. While this approach has been effective, it can also overlook protective surface antigens that are not abundant in virions produced by the vaccine virus.

Influenza virions possess two major surface antigens, hemagglutinin (HA or H) and neuraminidase (NA or N), that are both capable of eliciting a protective antibody response against influenza virus infections [2–5]. However, the HA amounts in the viral envelope are generally much higher than the NA amounts and can differ between influenza virus strains [6–8]. One contributing factor to the HA to NA ratio in a virion is the requirement to balance the low affinity receptor binding function of HA with the receptor removal function of NA [9–12]. This balance requirement continues to complicate the inclusion of NA in viral-based vaccines, as the ratio in virions is likely a unique trait for each HA and NA pair and current influenza vaccines are recommended to contain four different HA and NA pairs, two from influenza A viruses (IAVs) and two from influenza B viruses [13]. The two IAV antigen pairs are from representative field strains for the H1N1 and H3N2 subtypes that are annually chosen by the WHO based on surveillance data combined with serological and sequence analysis of the HA antigen.

In the U.S., viral-based influenza vaccines are the most commonly administered form of the vaccine and these are derived from inactivated virions produced in eggs, or more recently in cells [13]. To optimize the yields, viral-based influenza vaccine manufacturers generally use candidate vaccine viruses (CVVs), which are generated by reassortment of the representative HA and NA antigens with a non-pathogenic, high-growth influenza genome backbone such as the one from the A/PR8/1934 (PR8) strain [14]. Despite containing NA, CVVs are benchmarked against the wild-type field strain based on HA content, potentially resulting in the selection of strains that possess low NA amounts. While the underlying cause remains unclear, numerous studies have shown that influenza vaccines elicit a suboptimal NA antibody response [15–17], leading to the speculation that HA is immunodominant, or that NA amounts in the vaccine are insufficient or of poor quality.

The IAV genome is comprised of eight single-stranded influenza RNA gene segments, which encode for one or more viral proteins [18]. Several studies have used reverse genetics to change the virion incorporation of HA or NA by altering the promoter region, codons, or N-linked glycosylation sites in their respective gene segment [7,19–21]. Similar investigations have found that exchanging the internal gene segments can also affect the NA and HA amounts in virions [22–25], a trait supported by more mechanistic analysis of NA and HA expression in cells [26,27]. Here, we investigated if the PR8 influenza vaccine virus backbone can be modified to increase the NA virion content and balance the HA and NA antibody responses while preserving both antigens. In addition to the HA gene segment, our results demonstrate that the NA virion content is also dependent on the viral polymerase internal gene segments that are part of the virus backbone. The properties corresponding with the increased NA virion content are presented along with data showing that the inactivated virions elicit a stronger NA antibody response in mice without an associated loss in the HA antibody response. The mechanistic and translational implications of these results for producing viral-based vaccines that elicit more balanced NA and HA antibody responses are discussed.

## Results

### NA production by H1N1 influenza viruses in MDCK cells and eggs is strain dependent

**Fig 1:**
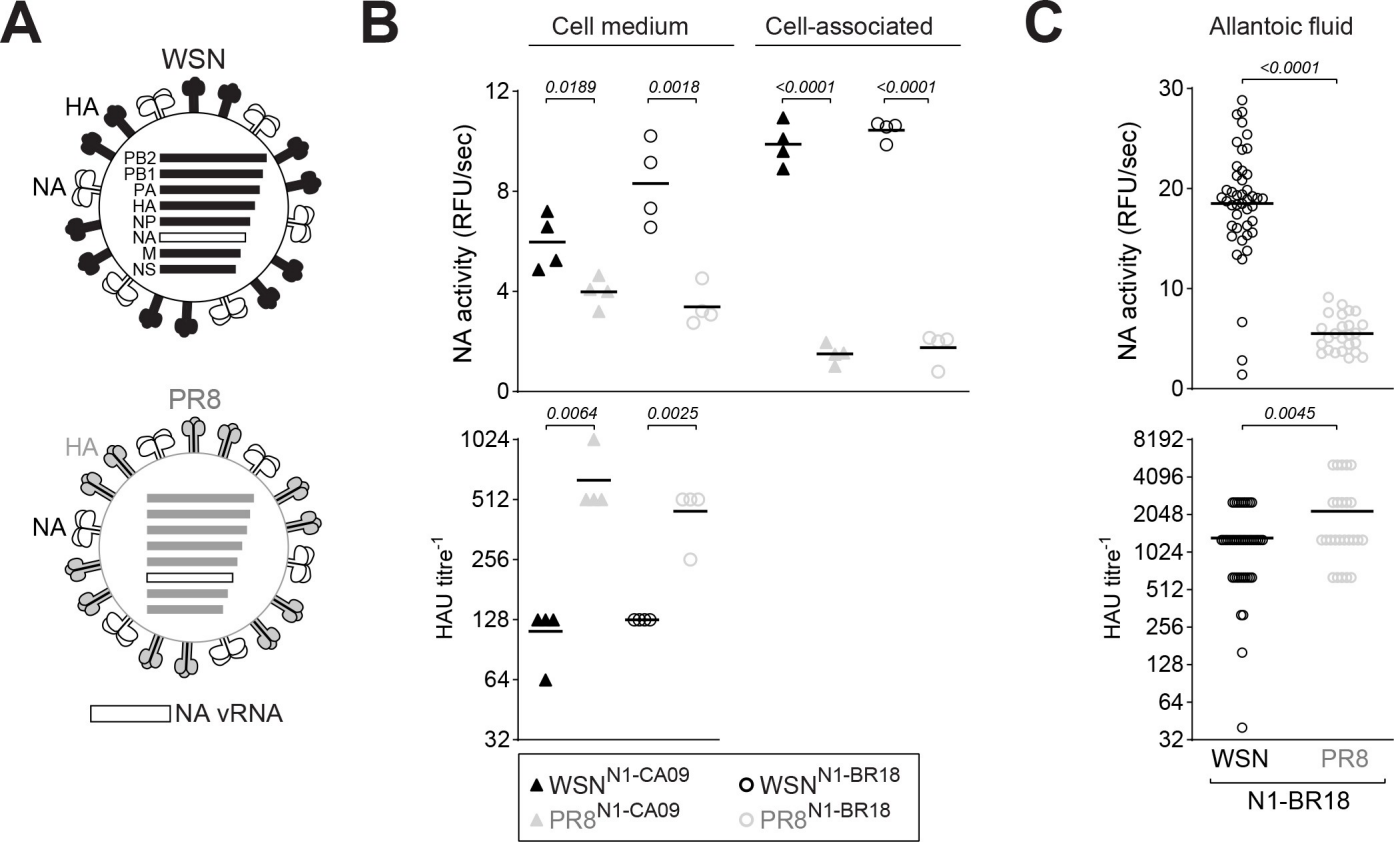
NA production by single-gene IAV reassortants is backbone-dependent. **A.** Diagram of the NA single-gene reassortant IAVs with a WSN and PR8 backbone. Viral RNA (vRNA) gene segments unique to each virus are color coded, including the HA and NA surface antigens. **B.** NA activity and HAU titres were measured from MDCK cells infected with the indicated viruses at a MOI of ~0.001. Upon completion of the infection, the medium and cell remains were collected, separated by centrifugation, and analyzed using equal sample amounts. Data from 4 independent biological replicates are displayed with the mean (bar). *P* values, calculated from a two-tailed unpaired t-test (95% CI), are shown. **C.** Eggs were harvested three days post-infection with the indicated viruses and the HAU titres and NA activity were measured using equal allantoic fluid volumes. Individual egg data from three independent experiments with different egg batches are shown with the mean (bar). *P* values were calculated from a two-tailed unpaired t-test (95% CI).

During previous studies, we observed that single-gene reassortant IAVs using NAs from subtype 1 (N1) are more easily rescued with a backbone from the A/WSN/1933 (WSN) strain compared to others and produce high NA levels [19,28,29]. To investigate this observation in a more controlled manner, single-gene reassortant IAVs carrying N1 antigens from the WHO recommended CVVs A/California/07/2009 (N1-CA09) or A/Brisbane/02/2018 (N1-BR18) were rescued using the seven gene segment backbone from WSN and PR8 (Fig 1A). PR8 was chosen for comparison because it is highly egg-adapted, like WSN, and often used to generate CVVs with high HA yields [14,30]. Initially, MDCK cells were infected with the four viruses at equal MOIs and the NA amount produced by each virus was analysed when the infection reached completion (CPE was ~100%). Both viruses with the WSN backbone displayed higher NA sialidase activity in the virus-containing cell medium and in the infected cell remains, indicating higher NA amounts were produced (Fig 1B, top panel). In contrast, viruses with the PR8 backbone generated higher hemagglutination unit (HAU) titres in the cell medium (Fig 1B, bottom panel). Similar results were obtained from embryonated eggs, as the WSN^N1-BR18^ virus produced higher NA levels in the allantoic fluid and lower HAU titres than the PR8^N1-BR18^ virus (Fig 1C), indicating the differences in the NA levels are likely due to strain specific viral gene products.

### Higher NA activity levels correlate with increased NA virion incorporation

**Fig 2:**
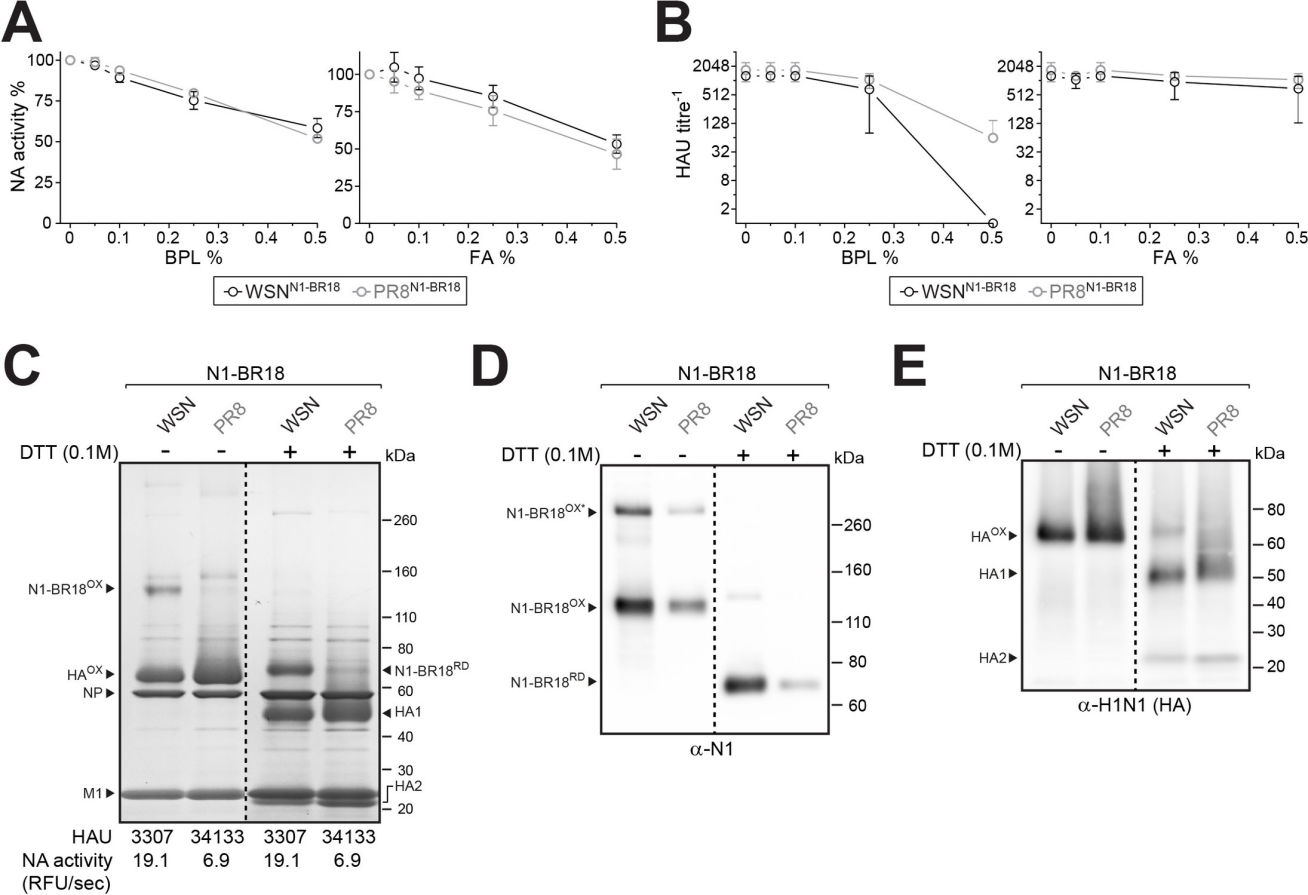
Egg produced WSN^N1-BR18^ virions incorporate more NA than PR8^N1-BR18^ virions. **A** and **B.** Graphs showing viral NA activity (**A**) and HAU titres (**B**) of the indicated viruses after treatment with increasing concentrations of β-propiolactone (BPL) and formaldehyde (FA). Treatment was done in allantoic fluid overnight at 4°C. NA activity of the untreated sample was set to 100%. Data from three independent biological replicates are displayed as the mean (circle) ± SD (error bars). **C.** Representative Coomassie stained gel of the purified BPL inactivated virions (5 μg per lane), untreated or reduced with DTT prior to resolution on a 4–12% SDS-PAGE gel. Oxidized (OX) and reduced (RD) forms of NA (N1-BR18) and HA are indicated with the viral proteins NP and M1. Note HA resolves as two polypeptides (HA1 and HA2) after reduction (+DTT). HAU titres and NA activity values are means from 3 independently purified batches with equal protein concentrations (1 mg/ml). **D** and **E.** Representative NA (**D**) and HA (**E**) immunoblots of the purified virions (0.5 μg per lane), untreated or reduced with DTT prior to SDS-PAGE. Oxidized and reduced NA and HA bands are indicated. (**D**) Asterisk indicates SDS resistant NA homo-tetramers that resolve under non-reducing conditions. (**E**) Note the H1N1 antisera has some reactivity towards NA.

To investigate if the increased NA levels reflect higher virion incorporation, we set out to purify inactivated WSN^N1-BR18^ and PR8^N1-BR18^ viruses from eggs. CVVs for egg-based vaccines are commonly inactivated with either beta-propiolactone (BPL) or formaldehyde (FA) [1,31], which can modify viral proteins in addition to ribonucleic acids [32–35]. Therefore, we first examined if BPL and FA affect the viral NA activity or HAU titre. BPL and FA caused similar concentration-dependent decreases in the allantoic fluid NA activity with FA resulting in slightly more variability (Fig 2A). In contrast, HAU titres significantly decreased with high concentrations of BPL (Fig 2B), especially WSN^N1-BR18^, suggesting the HA in this virus is more susceptible to BPL modification or the decrease in pH caused by BPL addition. Following confirmation of viral inactivation by three passages in eggs, we proceeded with 0.05% BPL because it showed little impact on NA activity and HAU titres, has a short half-life, and reacts with water to produce a non-toxic compound [32].

After BPL inactivation and purification, the viruses were adjusted to equal total protein concentrations and analysed. On both Coomassie stained SDS-PAGE gels (Fig 2C) and immunoblots (Fig 2D), bands at the expected molecular weight of oxidized NA (dimers) and reduced NA (monomers) [19,36] were more apparent for WSN^N1-BR18^ than PR8^N1-BR18^, indicating WSN^N1-BR18^ virions incorporate more NA. The increase in NA levels correlated with a decrease in HA content but did not affect the HA processing to HA1 and HA2 or the content of the viral nucleoprotein (NP) or matrix (M1) protein (Fig 2C and 2E). Supporting these observations, WSN^N1-BR18^ virions possessed ~3 times more NA activity, but ~10 times lower HAU titres than the PR8^N1-BR18^ virions (Fig 2C). These data indicate that one or more WSN backbone gene product supports higher viral incorporation of recent N1s at the expense of reduced HA levels.

### HA and the viral polymerase can influence NA virion incorporation

**Fig 3:**
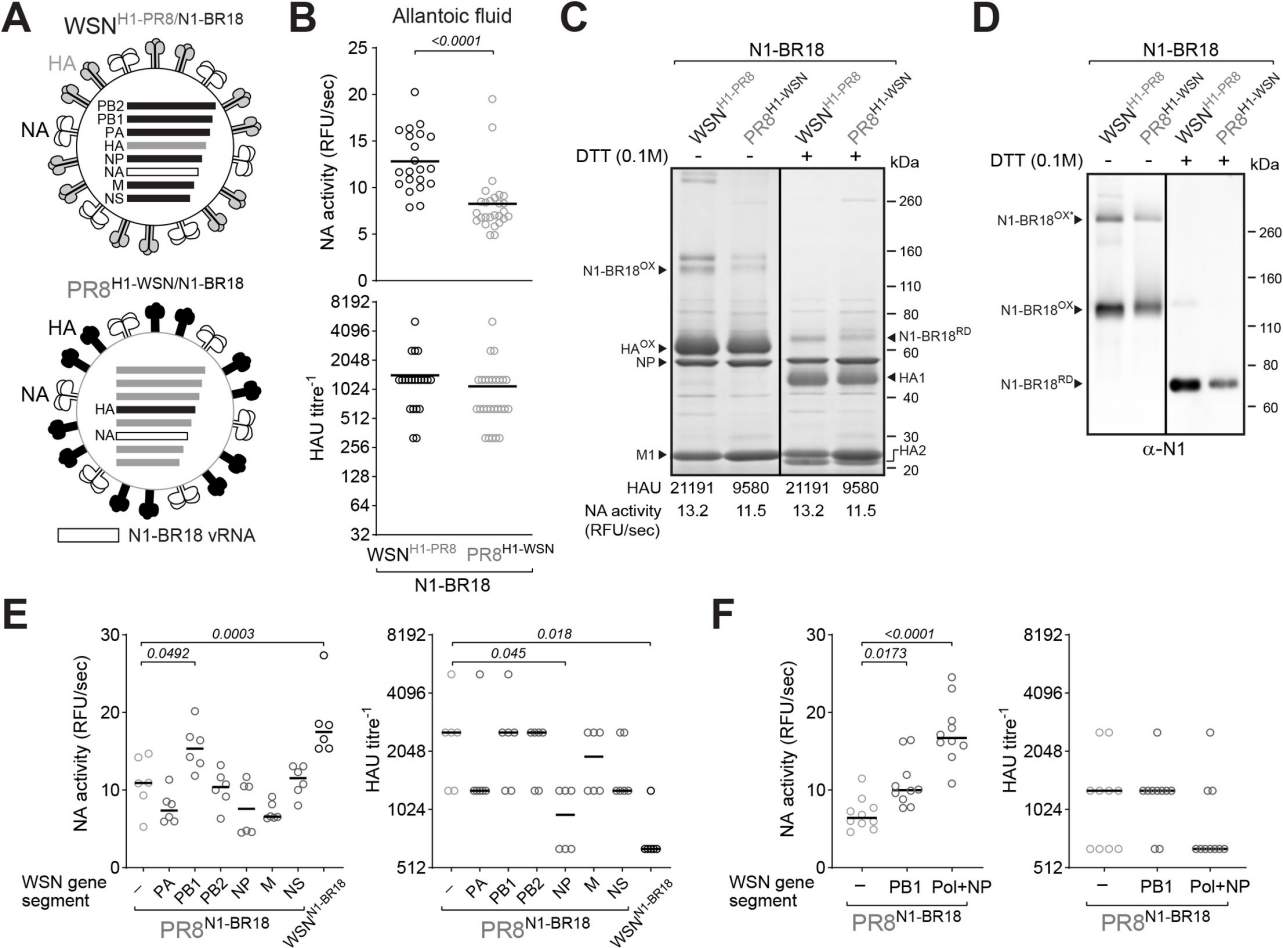
HA and the viral polymerase can influence NA virion incorporation. **A.** Diagram of the HA and NA double-gene reassortant IAVs. The NA gene segment (N1-BR18) is the same, whereas the HA gene segments from WSN and PR8 were swapped. **B.** Eggs infected with the indicated viruses were harvested 3 days post-infection and the HAU titres and NA activities were measured using equal allantoic fluid volumes. Individual egg data from three independent experiments with different egg batches are shown with the mean (bar). Significant *P* values, calculated from a two-tailed unpaired t-test (95% CI), are displayed. **C.** Coomassie stained gel showing 5 μg of the indicated, purified, BPL inactivated virions that were untreated or reduced with DTT prior to resolution by SDS-PAGE. Oxidized (OX) and reduced (RD) forms of N1-BR18 and HA are indicated with the viral proteins NP and M1. HAU titres and NA activity values are means from three independently purified virus batches with equal protein concentrations (1 mg/ml). **D.** NA immunoblots of the BPL inactivated purified virions (0.5 μg per lane) untreated or treated with DTT prior to SDS-PAGE. Oxidized and reduced N1-BR18 bands are indicated. Asterisk denotes SDS resistant N1-BR18 homo-tetramers. **E** and **F**. Eggs infected with the PR8^N1-BR18^ reassortant viruses containing the indicated WSN gene segments were harvested 3 days post-infection and the HAU titres and NA activity were measured using equal allantoic fluid volumes. Individual egg data from one egg batch are shown with the mean (bar). (**E**) WSN^N1-BR18^ included as a control. (**F**) Pol refers to PA+PB1+PB2 from WSN. Significant *P* values calculated by a one-way ANOVA using PR8^N1-BR18^ as a reference are displayed.

The two single-gene reassortant virus backbones (WSN and PR8) encode for distinct HAs raising the question of whether the observed increase in the virion NA content is dependent on HA due to potential changes in the functional balance. Therefore, we generated two double-gene reassortant viruses where the HA genes were swapped while the NA (N1-BR18) remained identical (Fig 3A). In eggs, the virus with the WSN backbone and the HA from PR8 (WSN^H1-PR8/N1-BR18^) produced higher NA levels and slightly better HAU titres than the virus with the HA from WSN and the PR8 backbone (Fig 3B). Following purification, bands corresponding to oxidized and reduced NA were apparent for both viruses on Coomassie stained gels (Fig 3C) and immunoblots (Fig 3D), which confirmed the NA levels were slightly higher in the WSN^H1-PR8/N1-BR18^ virions. The virion HA content was rather consistent, but somewhat lower M1 levels were observed in the WSN^H1-PR8/N1-BR18^ virions. In line with the Coomassie gel, WSN^H1-PR8/N1-BR18^ virions possessed ~15% more NA activity than PR8^H1-WSN/N1-BR18^ virions, whereas the HAU titres were more than double (Fig 3C). These results implied that HA and one or more WSN backbone gene products support increased viral incorporation of recent N1s and that the HA from PR8 generates higher HAU titres with turkey red blood cells than the HA from WSN.

To determine if a specific WSN backbone gene segment contributes to the higher NA virion incorporation, a series of PR8^N1-BR18^ reassortant viruses carrying a different WSN internal gene segment were created and tested in eggs. Only the virus containing the PB1 gene segment from WSN produced higher NA levels and it also showed no observable loss in HAU titres (Fig 3E), indicating the viral polymerase from WSN likely contributes to the higher NA content in the virions. A similar analysis of a PR8^N1-BR18^ reassortant virus carrying all the WSN polymerase subunits (PB1, PB2 and PA) together with NP, confirmed that the polymerase from WSN is largely responsible for the higher NA content in virions containing the WSN backbone (Fig 3F).

### Internal WSN backbone gene segments support high viral incorporation of recent N1s

**Fig 4:**
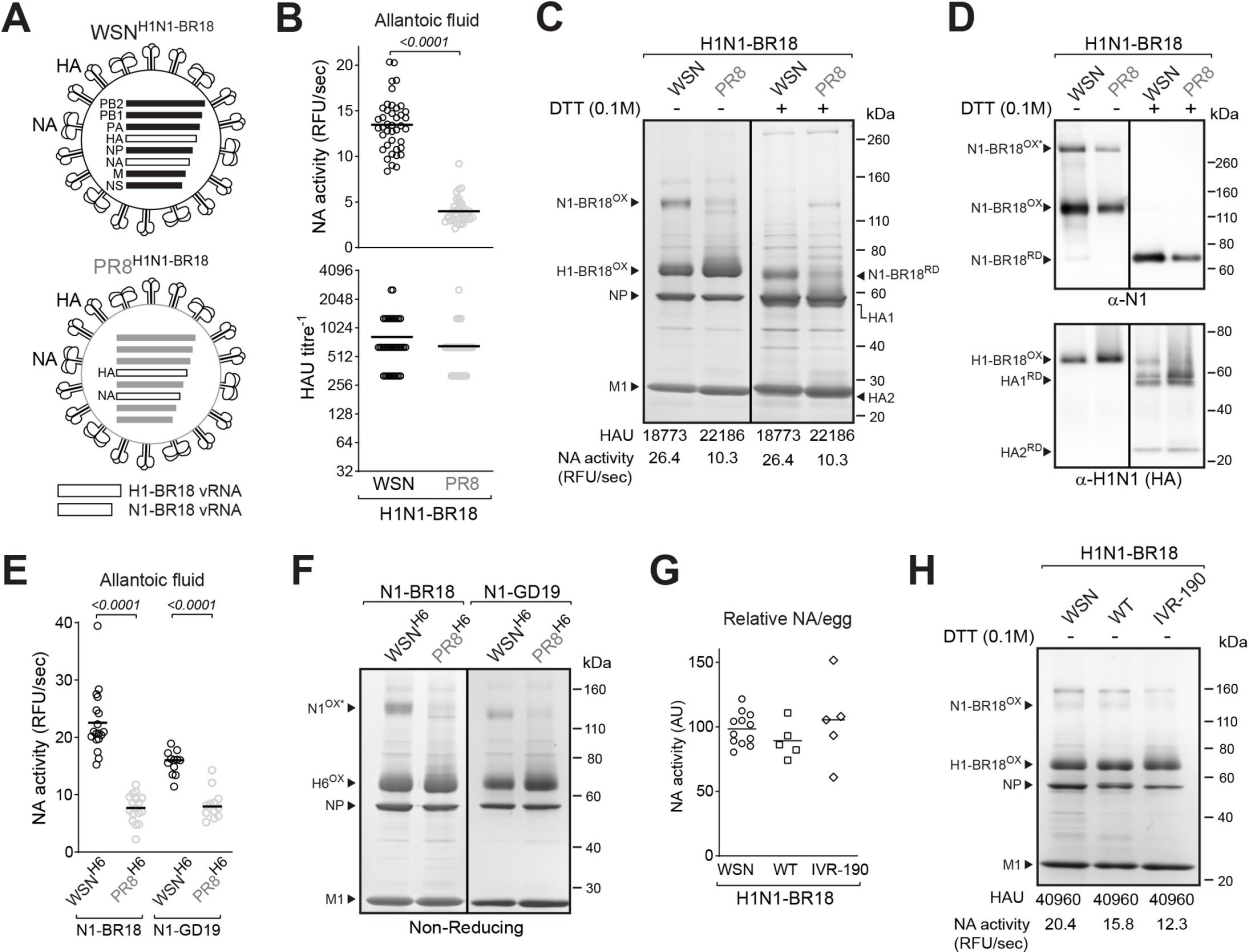
The WSN backbone supports N1 virion incorporation independent of HA. **A.** Diagram of the PR8 and WSN double-gene reassortant viruses with HA and NA gene segments from the H1N1 strain A/Brisbane/02/2018. **B.** Eggs were harvested 3 days post-infection and the HAU titres and NA activities were measured in each egg using equal allantoic fluid volumes. Individual egg data from three independent experiments with different egg batches are shown with the mean (bar). Significant *P* values, calculated from a two-tailed unpaired t-test (95% CI), are displayed. **C.** Coomassie stained gel of the indicated purified BPL inactivated virions (5 μg per lane), treated with DTT as indicated. Oxidized and reduced N1-BR18 and H1-BR18 are indicated with NP and M1. HAU titres and NA activity values are means from three independently purified virus batches with equal protein concentrations (1 mg/ml). **D.** NA (top) and HA (bottom) immunoblots of the purified BPL inactivated virions (0.5 μg per lane), untreated or reduced with DTT prior to SDS-PAGE. Oxidized and reduced N1-BR18 and H1-BR18 are indicated. Asterisk denotes SDS resistant NA homo-tetramers. Note the H1N1 antisera has some NA reactivity. **E.** Eggs were harvested 3 days post-infection with the indicated viruses and the NA activities were measured using equal allantoic fluid volumes. Individual egg data are shown with the mean (bar). *P* values are from a two-tailed unpaired t-test (95% CI). **F.** Non-reducing Coomassie stained gel containing the indicated purified virions (5 μg per lane). Oxidized forms of the N1s and H6 are indicated with NP and M1. **G.** Graph displaying the relative total NA amount from individual eggs infected with the indicated virus for three days. The mean (bar) is included. **H.** Coomassie stained gel showing the purified BPL inactivated virions (5 μg per lane). Oxidized and reduced forms of N1-BR18 and H1-BR18 are indicated with NP and M1. HAU titres and NA activities are from purified virus batches with equal protein concentrations (1 mg/ml).

To mitigate the HA influence on the NA virion content, two double gene-reassortant viruses were generated using the recommended H1 and N1 antigens for the 2019–2020 influenza season (Fig 4A). Again, the virus with the WSN backbone (WSN^H1N1-BR18^) produced higher NA levels in eggs than the virus with the PR8 backbone virus (PR8^H1N1-BR18^) and rather similar HAU titres (Fig 4B). Coomassie stained SDS-PAGE gels and activity measurements of the purified virions confirmed that the NA levels were significantly higher in the WSN^H1N1-BR18^ virions (Fig 4C). However, the NA increase coincided with a slight decrease in the HAU titres and the HA amounts observed on both Coomassie stained gels and immunoblots (Fig 4C and 4D). In a comparative analysis of reassortant viruses containing an unrelated HA subtype (H6 from the strain A/turkey/Mass/3740/1965) and either N1-BR18 or the NA from another vaccine strain A/Guangdong-Maonan/SWL1536/2019 (N1-GD19), the viruses with the WSN backbone still produced more NA activity in eggs (Fig 4E) and showed higher NA content (Fig 4F), demonstrating the WSN backbone generally supports the incorporation of recent N1s into virions.

### Changing the CVV backbone can provide advantages for NA virion content

Most seasonal viral-based influenza vaccines are produced in eggs using either the recommended wild type field strain or a CVV that has been generated with high growth properties [14]. Therefore, we benchmarked the NA levels produced by the WSN^H1N1-BR18^ virus against the wild-type (WT) BR-18 H1N1 field isolate as well as the BR18 CVV (IVR-190) that was approved for manufacturing egg-based influenza vaccines for the 2019–2020 season. Based on the relative NA activity per egg, the WSN^H1N1-BR18^ virus generated NA levels that were on par or better than the WT field isolate, but slightly lower than IVR-190 (Fig 4G). Following isolation and normalization for total protein levels, the NA content and activity in WSN^H1N1-BR18^ virions was found to be slightly higher than the WT strain and almost twice as high as IVR-190 (Fig 4H). However, it is worth noting that the total viral yields from the IVR190 strain were more than triple what was obtained from the WSN^H1N1-BR18^ strain. This data further shows that influenza viruses can accommodate a range of NA and HA ratios for a given pair and that the NA content in CVVs used to produce influenza vaccines can be increased.

### Plasticity in the HA and NA balance allows for increases in the NA virion incorporation

**Fig 5:**
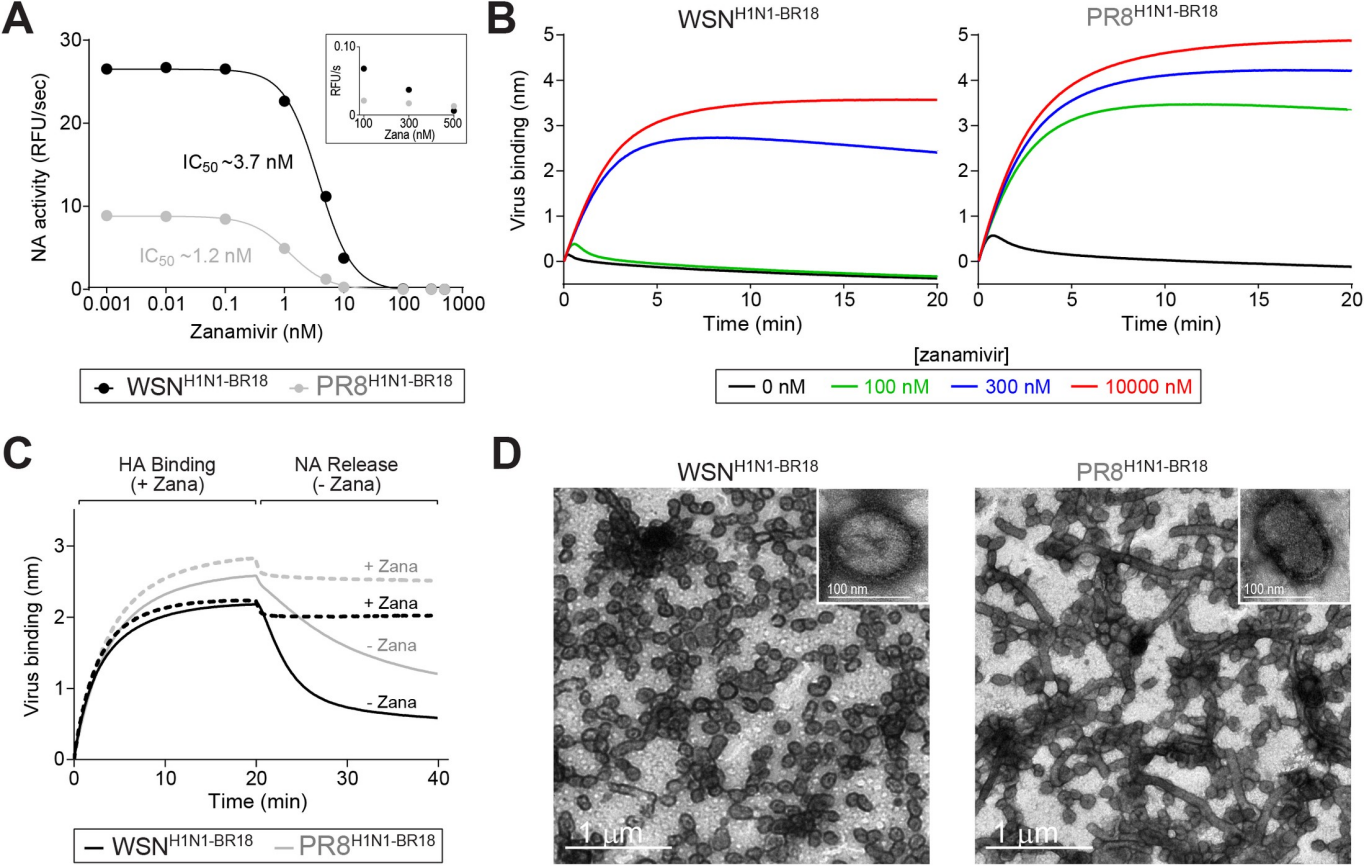
Property analyses of viruses with different NA to HA ratios. **A.** NA activity in the purified BPL inactivated viruses were measured using MUNANA in the presence of increasing zanamivir concentrations to calculate the 50% inhibitory concentration (IC_50_). Measurements were made using 0.5 μg of total viral protein. Inset shows the NA activity values at high zanamivir concentrations. **B.** Binding of purified WSN^H1N1-BR18^ virions (left graph) and PR8^H1N1-BR18^ virions (right graph) to 3’-SLN were measured in the presence of the indicated zanamivir concentrations by biolayer interferometry (BLI). Each binding curve was generated with a biosensor containing the same density of immobilized 3’SLN and ~0.1 mg/ml of purified virus. **C.** HA binding of the purified virions (~0.1 mg/ml) to 3’SLN and NA-mediated release of bound virus were measured by BLI. HA-binding was monitored for 20 min in the presence of 10 μM zanamivir to inhibit NA activity. The biosensor containing the bound virus was moved to a new well with (dashed line) or without zanamivir (line) and the NA-mediated release was followed. **D.** Negative stained transmission electron microscopy images of BPL inactivated purified WSN^H1N1-BR18^ and PR8^H1N1-BR18^ virions. Insets contain higher magnification images of a spherical virion. Scale bars (white) are included.

Changes in the NA to HA ratio suggests that the functional balance between a particular NA and HA pair in a virus has some level of plasticity. To investigate the functional balance, we examined the WSN^H1N1-BR18^ and PR8^H1N1-BR18^ viruses using the NA inhibitor zanamivir in conjunction with a bio-layer interferometer (BLI) equipped with a streptavidin biosensor containing a biotinylated 2,3-sialic acid analogue receptor, Neu5Ac-α2-3Gal-β1-4GlcNAc-β-PAA-biotin (3’SLN). Initially, we confirmed that the zanamivir 50% inhibitory concentration (IC_50_) for the purified BPL inactivated viruses were similar (Fig 5A) and that the IC_50_ values were in line with previous studies using H1N1 IAVs [37]. When the 3’SLN association was monitored by BLI, the HA-receptor binding of both viruses was disrupted by NA within a few seconds and the disruption could be overcome by zanamivir in a dose-dependent manner (Fig 5B). However, higher zanamivir concentrations were required for efficient 3’SLN binding of the WSN^H1N1-BR18^ virus (Fig 5B), presumably because the virus possesses more NA, requiring higher zanamivir concentration for complete NA activity inhibition (Fig 5A, inset).

The BLI results indicate that either the low HA content and/or higher NA content altered the HA-mediated binding of WSN^H1N1-BR18^ compared to PR8^H1N1-BR18^. To separate these two processes, HA-mediated binding to 3’SLN was monitored in the presence of high zanamivir concentrations and NA-mediated release was examined by removing the inhibitor [38]. In the presence of zanamivir, both viruses showed similar HA binding with the WSN^H1N1-BR18^ virus displaying lower saturation levels (Fig 5C). In contrast, the NA mediated release of the WSN^H1N1-BR18^ virus was substantially faster than the PR8^H1N1-BR18^ virus (Fig 5C), confirming that the increase in the NA virion content is reflected in a functional analysis. These results indicate that IAVs can support variations in the HA and NA ratio of a given pair that result in different functional balances.

### Viruses with the WSN backbone and higher NA content are predominantly spherical

NA has been proposed to cluster on the viral surface at regions of high membrane curvature [6,39,40]. Therefore, we examined the morphology of the purified BPL inactivated WSN^H1N1-BR18^ and PR8^H1N1-BR18^ virions by electron microscopy. The WSN^H1N1-BR18^ virions displayed a homogeneous spherical morphology with a diameter of ~100 nm, whereas the PR8^H1N1-BR18^ virions showed a mixture of spherical and filamentous morphologies, with the later having lengths greater than 1 μm (Fig 5D). While it is interesting to speculate that NA induced membrane crowding results in spherical virions with higher NA content, it is equally plausible that the morphology difference is an inherent property of the WSN backbone or a partial contributing factor to the recruitment of NA into the virions.

### Increasing the NA virion content can balance the NA and HA antibody response

**Fig 6:**
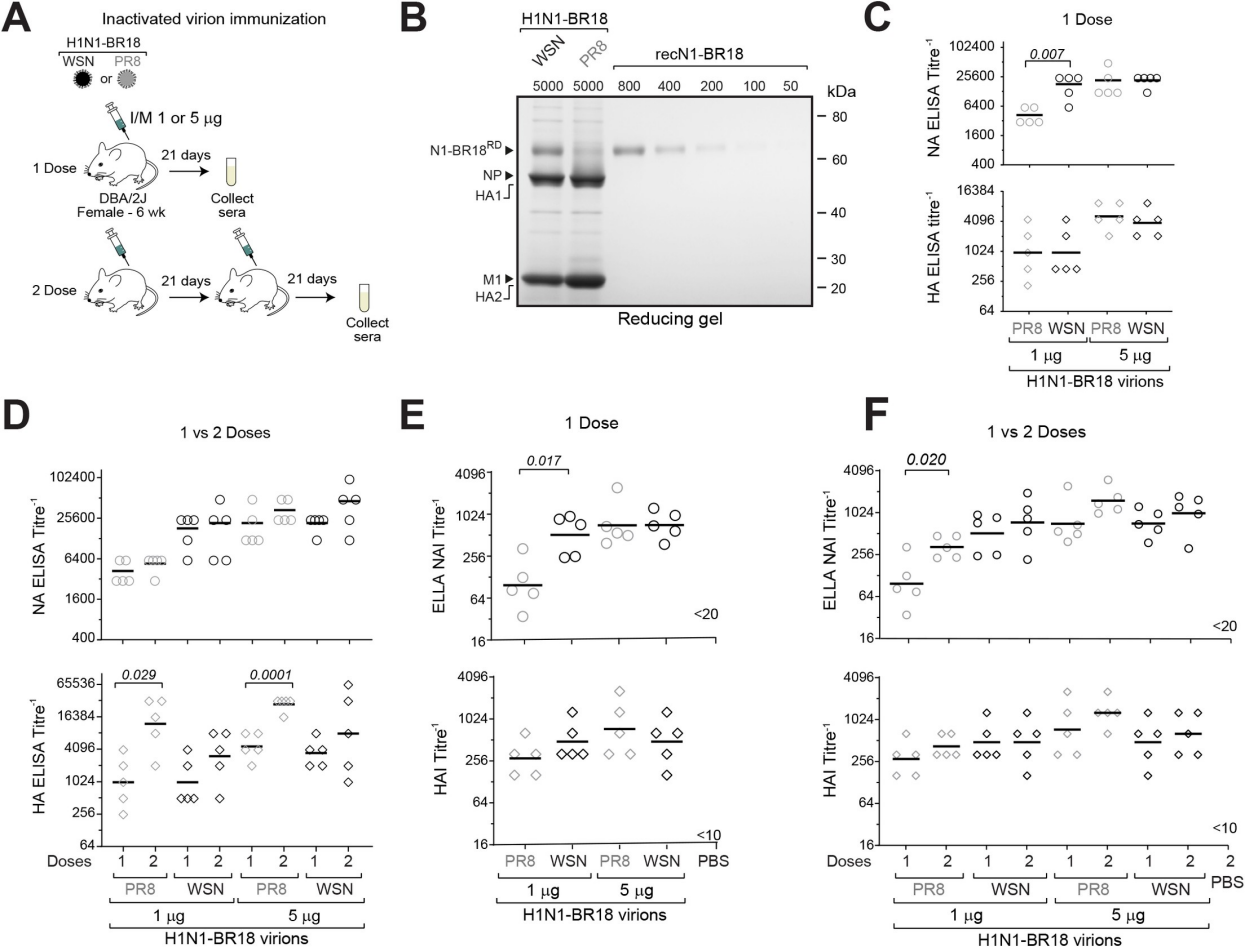
Higher NA virion content corresponds with a stronger NA antibody response. **A.** Diagram depicting the one and two dose immunization schemes in mice using two different amounts (1 μg and 5 μg) of the purified BPL inactivated WSN^H1N1-BR18^ and PR8^H1N1-BR18^ virions. **B.** Reducing Coomassie stained gel used to estimate the NA content in the purified BPL inactivated virions by densitometry. Recombinant N1-BR18 was used as a reference standard. **C.** Serum NA (Top) and HA ELISA titres (Bottom) obtained from mice following 1 dose of the indicated virion amounts are displayed. *P* values comparing the different virions at each dose were calculated by a two-tailed unpaired t-test (95% CI). **D.** Graphs showing the serum NA (Top) and HA ELISA titres (Bottom) obtained from mice following one or two doses of the indicated virion amounts. *P* values comparing one and two doses for each virion and protein amount were calculated by a two-tailed unpaired t-test (95% CI). **E.** ELLA NAI titres (Top) and the HAI titres (Bottom) obtained from mice following one dose with the indicated virion amounts are displayed. *P* values comparing the different virions at each dose were calculated by a two-tailed unpaired t-test (95% CI). **F.** Graphs comparing the ELLA NAI titres (Top) and HAI titres (Bottom) in the mice that received one and two doses of the indicated virion amounts. *P* values comparing one and two doses for each virion and protein amount were calculated by a two-tailed unpaired t-test (95% CI).

To determine if increasing the NA virion content can improve the NA antibody response, a comparative immunization analysis was performed using mice (Fig 6A). Prior to the immunization experiment, the amount of NA in the inactivated purified WSN^H1N1-BR18^ and PR8^H1N1-BR18^ virions was estimated by Coomassie stained gels using recombinant N1-BR18 protein as a standard (Fig 6B). Based on the densitometry analysis, 5 μg of total WSN^H1N1-BR18^ and PR8^H1N1-BR18^ viral protein were estimated to contain ~750 ng and ~225 ng of N1-BR18 protein, respectively, a ratio in-line with the NA activity difference (Figs 6B and 4C). Mice were then intramuscularly immunized with 1 or 5 μg of purified inactivated WSN^H1N1-BR18^ or PR8^H1N1-BR18^ virions in parallel. One cohort received one dose while the other received a second dose 21 days later and sera were collected 21 days following the last immunization.

Mice that received a single 1 μg dose of WSN^H1N1-BR18^ virions generated ~4-fold higher NA antibody binding titres than mice that received 1 μg of PR8^H1N1-BR18^ virions, and the titres were similar to those from mice that received one 5 μg dose of either virion (Fig 6C, top). In contrast, the HA antibody binding titres from all the mice that received a single 1 μg dose of virions were almost identical and the titres increased similarly at the 5 μg dose level for both virions (Fig 6C, bottom). The second dose induced subtle but insignificant increases in the NA antibody binding titres, whereas the HA antibody binding titres showed a more pronounced benefit from the second dose, especially for the mice immunized with the PR8^H1N1-BR18^ virions (Fig 6D).

We also determined the hemagglutination inhibition (HAI) and NA inhibition (NAI) titres as these are likely to be more indicative of protection [41,42]. Similar to the ELISA results, the single 1 μg dose of WSN^H1N1-BR18^ virions induced NAI titres that were ~5-fold higher than those from a single 1 μg dose of PR8^H1N1-BR18^ virions and the titres were on par with those from mice that received one 5 μg dose of either virion (Fig 6E, top). In contrast to the ELISA results, the HAI titres were relatively similar across the groups (Fig 6E, bottom). The second dose only showed a significant increase in the NAI titres for the 1 μg of PR8^H1N1-BR18^ virions, which contained the least amount of NA protein (Fig 6F top), and it induced slight but insignificant increases in the HAI titres (Fig 6F, bottom).

**Fig 7:**
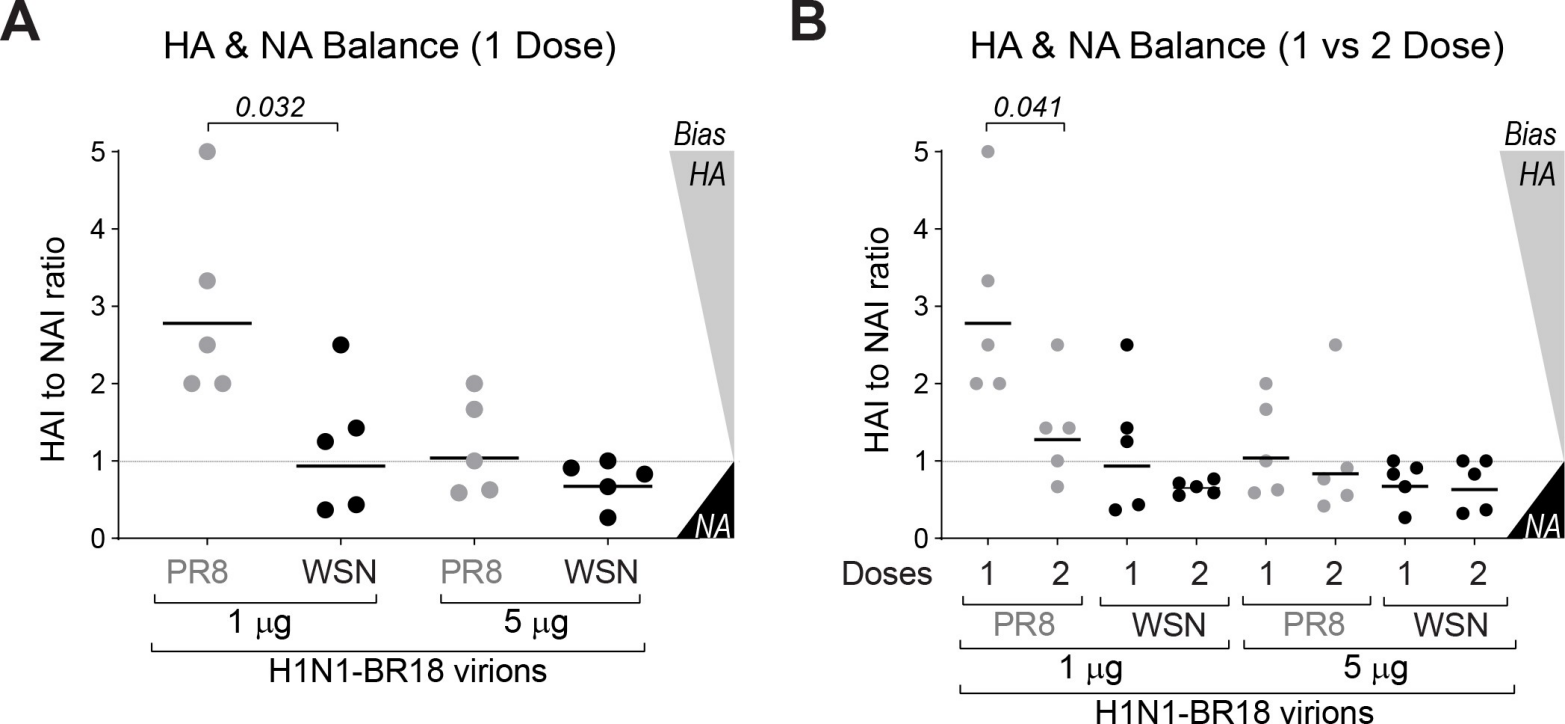
Virions with increased NA content elicit a more balanced NA and HA response. **A.** The HAI to NAI titre ratio was plotted for each serum from the mice that received one dose of the indicated virion amount. Values >>1 indicate the antibody response is HA biased, values ~1 indicate a more balanced response and values <<1 indicate a NA biased response. *P* values comparing the different virions at each dose were calculated by a two-tailed unpaired t-test (95% CI). **B.** Graph comparing the HAI to NAI titre ratio for each serum from the one and two dose immunizations with the indicated virion amounts. *P* values comparing one and two doses for each virion and protein amount were calculated by a two-tailed unpaired t-test (95% CI).

Plotting the HAI to NAI ratio from each serum in the single dose immunization groups revealed a clear trend toward a more balanced response with increasing NA virion amounts as the ratios coalesced to 1 (Fig 7A). A similar observation was made with respect to increasing NA virion amounts following the second 1 μg dose. For the rPR8^H1N1-BR18^ virions, which contained the lowest NA amount, the ratios decreased from ~2.5 (HA biased) to ~1.25 (more balanced) after the second 1 μg dose, and for the WSN^H1N1-BR18^ virions the ratios coalesced to slightly below 1 after the second 1 μg dose (Fig 7B). Together, these results imply that attainable increases in NA virion content can improve the balance of the NA and HA inhibitory antibody responses, especially at lower antigen concentrations, indicating this potentially beneficial response can be achieved without dramatically increasing the HA content in a viral-based vaccine dose.

## Discussion

Influenza NA is a labile, homotetrameric Ca^2+^-dependent enzyme with a complex maturation process that is coordinated by the N-terminal transmembrane region and the C-terminal enzymatic head domain [28,36,43–46]. Recently, it was shown that each NA monomer in a tetramer functions independently (noncooperatively) and that the low stability of the Ca^2+^-dependent oligomeric conformation varies between strains of the same subtype [29]. These nonideal antigen properties combined with the limited abundance, likely impact both the quantity and quality of the NA in viral-based influenza vaccines, hindering a productive antibody response [15–17]. As influenza vaccine efficacy remains less than ideal, there are significant efforts to enhance the NA response to create vaccines that provide higher efficacy and broader cross protection against circulating strains [47].

Our results demonstrate that the bottleneck of NA abundance in H1N1 virions can be addressed by changing the backbone or the polymerase gene segments in the CVVs. This approach preserves the NA and HA antigens and the increase in NA content induced higher NAI titres without significantly reducing the HAI titres, resulting in a more balanced NA and HA antibody responses. The improved balance against both antigens was more evident in the single immunizations with the lowest virion amounts, suggesting the initial NA antibody response in mice is limited by a quantity threshold rather than HA immunodominance. Future challenge experiments using immunizations with lower virion amounts will help to determine if the increase in NA content can decrease the antigen amount required for protection, and to define the NA antigen amounts that correlate with protection from strains with unmatched HAs.

Although WSN is a commonly used highly adapted lab strain like PR8, it has not received much consideration as a CVV backbone because of many reports showing it is neurovirulent in mice [48–50]. However, the neurovirulence trait has been linked to the NA in WSN [51,52], requires intracerebral inoculation [48], and in CVVs both the HA and NA gene segments are replaced during generation. In addition, we have observed no clinical symptoms (*e*.*g*. fever, weight loss, nasal symptoms or low energy) in ferrets that were inoculated with a series of identical HA and NA double-gene WSN and PR8 reassortants for another study and our results suggest polymerase components alone may be sufficient. We are not certain that using classical reassortment to create a CVV with a WSN backbone will improve viral growth and retain the higher NA virion content, but our results showing that the NA virion content is higher than an approved CVV and the viral protein yields are on par with the WT field isolate that is used to benchmark related CVVs, suggest further investigation is warranted.

The neurovirulence requirement for the NA from WSN has been attributed to its unique ability to enable trypsin-independent viral replication by facilitating the recruitment of the protease plasminogen for HA processing [53,54]. Although not required, the M and NS gene segments from WSN were also shown to promote viral growth in neurons [51,52], which possess especially high sialic acid content [55], suggesting these segments could potentially contribute to the increased NA virion content. In line with this possibility, the virions with the WSN backbone displayed a more homogeneous spherical morphology, a phenotype that has previously been associated with the M gene segment [56–58]. Mechanistically, spheres could potentially accommodate more copies of asymmetrical shaped NA tetramers which have been proposed to localize to membrane regions with high curvature [39,40,58]. A second difference was observed during purification where the WSN virion pellet showed a propensity to flocculate and foam during resuspension, indicating potential lipid differences between the virions. Despite these observations, the data from eggs indicates that the viral polymerase, especially the PB1 subunit, is largely responsible for increasing the content of recent N1s in WSN virions. This raises the questions of whether the increased NA is due to a more optimal NA promoter polymerase pairing in WSN and if other PB1s from seasonal IAVs could be used to produce virions with even higher NA amounts. To gain more insight into these questions studies analyzing the PB1 residues that differ between the two strains are needed.

While our data indicate that virions can support a range of functional balances for the same NA and HA pair, the influence of HA on the NA content in a vaccine strain is unavoidable and an upper limit for the NA content in a virion likely exists. The HA content decrease that coincides with the increase in the NA virion content is a rather expected outcome due to mass action and the packaging limitations of a virion, however it is also not ideal for maximizing the vaccine dose number unless the added benefit from NA can improve the efficacy and/or decrease the required dose amount. These critical questions emphasize the need for a systematic analysis of the requirements for protection by NA and HA independently, and together, to determine if the theoretical upper limit for NA in a virion is enough to overcome the NA quantity bottleneck in current influenza vaccines that are dosed based on HA amounts.

## Materials and methods

### Ethics statement

All animal experiments were approved by the U.S. FDA Institutional Animal Care and Use Committee (IACUC) under Protocol #2003–18. The animal care and use protocol meets National Institutes of Health (NIH) guidelines.

### Reagents and antibodies

Dulbecco’s Modified Eagles Medium (DMEM), fetal bovine serum (FBS), L-glutamine, penicillin/streptomycin (P/S), Opti-MEM I (OMEM), anti-goat IgG HRP-linked secondary antibody, Simple Blue Stain, Novex 4–12% Tris-Glycine SDS-PAGE gels, dithiothreitol (DTT) Immulon-2HB 96-well plates, Nickel coated 96-well plates and Lipofectamine 2000 transfection reagent were all purchased from Thermo Fisher Scientific. Zanamivir and 2’-(4-methylumbelliferyl)-α-d-*N*-acetylneuraminic acid (MUNANA) were acquired from Moravek Inc and Cayman Chemicals, respectively. β-propiolactone, formaldehyde, *o*-Phenylenediamine dihydrochloride (OPD), and anti-mouse IgG HRP linked secondary were purchased from Sigma. Anti-rabbit IgG HRP-linked secondary antibody and 0.45-μm polyvinylidene difluoride (PVDF) membrane were obtained from GE healthcare. Specific-Pathogen-Free (SPF) eggs and turkey red blood cells (TRBCs) were purchased from Charles River Labs and the Poultry Diagnostic and Research Center (Athens, GA), respectively. The H1N1 A/Brisbane/02/2018 field isolate (WT) and CVV (IVR-190) were kindly provided by the WHO. Rabbit Antisera against NA was generated by Agrisera (Sweden) using NA-WSN residues 35–453 isolated from *E*. *coli* inclusion bodies [29]. Polyclonal goat antiserum against the H1N1 influenza virus A/Fort Monmouth/1/1947 (NR-3117) was obtained from BEI Resources, NIAID, NIH.

### Plasmids and constructs

The eight reverse genetics (RG) plasmids encoding the WSN33 and PR8 gene segments were provided by Dr. Robert Webster (St. Jude Children’s Research Hospital). The RG plasmids were sequenced before use and correspond with the following GenBank Identifications: LC333182.1 (WSN33-PB2), LC333183.1 (WSN33-PB1), LC333184.1 (WSN33-PA), LC333185.1 (WSN33-HA), LC333186.1 (WSN33-NP), LC333187.1 (WSN33-NA), MF039638.1 (WSN33-M) LC333189.1 (WSN33-NS), CY038902.1 (PR8-PB2), CY038901.1 (PR8-PB1), CY084019.1 (PR8-PA), CY146825.1 (PR8-HA), CY038898.1 (PR8-NP), CY038897.1 (PR8-NA), MH085246.1 (PR8-M), and CY038899.1 (PR8-NS). The RG plasmids containing the NA (N1-CA09) and the H6 gene (A/turkey/Mass/3740/1965) were described previously [28,59]. To generate the NA (N1-BR18) and HA (H1-BR18) RG plasmids, the NA and HA gene segments from IVR-190, grown in SPF chicken eggs, were amplified by RT-PCR and cloned into the pHW2000 plasmid using restriction enzymes [60]. All constructs were confirmed by sequencing (FDA core facility or Macrogen).

### Cell culture and viral reverse genetics

Madin-Darby canine kidney 2 (MDCK.2; CRL-2936) cells and HEK 293T/17 cells (CRL-11268) were both obtained from LGC Standards and cultured at 37°C with 5% CO_2_ and ~95% humidity in DMEM containing 10% FBS and 100 U/ml P/S. Reassortant viruses were created by 8-plasmid reverse genetics [60] in 6-well plates using the indicated NA, or NA and HA pair, and the complimentary seven, or six, backbone gene segments of WSN or PR8. For each virus, ~1 x 10^6^ 293T and ~1 x 10^6^ MDCK.2 cells were plated in a well the day before. The next day, the eight plasmids (1 μg of each) were added to 200 μl of OMEM, mixed with 18 μl of lipofectamine, and incubated 45 min at room temperature. After the incubation, the cells were washed with 2 ml OMEM, the mixture was added to the well and incubated 5 min at 37°C before receiving 800 μl OMEM. At ~24 h post-transfection, 1 ml OMEM containing 4 μg/ml TPCK trypsin was added to each well. Rescued viruses in the culture medium were harvested ~96 h post-transfection, clarified by sedimentation (2000 × g; 5 min) and passaged using SPF eggs or MDCK.2 cells.

### Viral passaging and analysis in MDCK cells

Reverse genetics rescued viruses in culture medium were diluted 1:1,000 in infection media (DMEM with 0.1% FBS, 0.3% BSA, 1% P/S and 2 μg/ml of TPCK trypsin) and 10 ml was added to a T75cm flask containing MDCK.2 cells at ~95% confluency. The cells were incubated 3 days at 37°C prior to harvesting. The virus-containing culture medium was clarified by centrifugation (2,000 x g; 5 min), aliquoted, stored at -80°C, and the median tissue culture infectious dose (TCID_50_ ml^−1^) was determined by cytopathic effects (CPE) in MDCK.2 cells at 72 h [61]. For the NA expression analysis, T25cm flasks with ~95% confluent MDCK.2 cells were infected with the viruses at an MOI ~0.001 and processed as follows. Cells were incubated with virus diluted in infection media for 30 min at 4°C. Unbound virus was removed and 5 ml of fresh infection media was added. Infections were monitored by observing CPE and the viruses and cells were harvested by scraping once the infection completed, ~3 days. The cell remains were isolated by sedimentation (5 min at 10,000 x g), virus containing media was transferred to a new tube and the cell pellet was lysed in 500 μl of lysis buffer (20 mM Tris, pH 7.4, 150 mM NaCl, 1% *n*-dodecyl *β*-D-maltoside, 1X protease inhibitor (Sigma)). Lysates were sonicated on ice 30 s, sedimented (20,000 x *g*, 5 min), and the post-nuclear supernatants were retained. NA activity was measured using 50 μl of medium and 5 μl of the post nuclear supernatant, whereas HAU titres were determined using 50 μl of medium.

### Viral passaging in SPF chicken eggs

Initial passages (E1) were carried out by inoculating 9–11 day old embryonic SPF chicken eggs with 100 μl of the rescued virus in culture medium. Eggs were incubated for 3 days at 33°C and placed at 4°C for 2 h prior to harvesting the allantoic fluid. The allantoic fluid was clarified by centrifugation (2000 x g; 5 min), aliquoted and stored at -80°C. The second passage was carried out similarly. E1 aliquots of the viruses being compared were thawed at 37°C, diluted 1:1000 in sterile PBS and 100 μl was used to inoculate groups of eight or more 9–11 day old embryonic eggs. The allantoic fluid was harvested individually from each egg at 3 days post-infection, clarified by centrifugation (2000 x g; 5 min), and NA activity and HAU measurements were taken prior to inactivation and purification of the virions.

### Viral inactivation by β-propiolactone and formaldehyde

Aliquots (1 ml) of the WSN^N1-BR18^ and PR8^N1-BR18^ viruses in allantoic fluid were treated with the indicated concentrations of β-propiolactone (BPL) or formaldehyde (FA) for ~16 h at 4°C. Following the treatment, NA activity was measured in each sample using 10 μl of allantoic fluid and HAU titres were measured using 50 μl of allantoic fluid. For inactivation prior to purification, viruses in allantoic fluid were treated with 0.05% BPL for ~16 h at 4°C.

### Viral purification

BPL treated viruses in allantoic fluid were first isolated by sedimentation (100,000 x g; 45 min) at 4°C through a sucrose cushion (25% w/v sucrose, PBS pH 7.2 and 1 mM CaCl_2_) equal to 12.5% of the sample volume. The pelleted virions were resuspended in 500 μl sucrose 12.5% w/v in PBS pH 7.2 containing 1 mM CaCl_2_, layered on top of a discontinuous sucrose gradient containing four 8.5 ml sucrose layers (60% w/v, 45% w/v, 30% w/v and 15% w/v in PBS pH 7.2 and 1 mM CaCl_2_) and centrifuged at 100,000 x g for 2 h at 4°C. Fractions were collected and the density was determined using a refractometer. Fractions with a density between 30–50% w/v sucrose were pooled, mixed with 2 volumes of PBS pH 7.2 and 1 mM CaCl_2_, and sedimented (100,000 x g; 45 min). The supernatant was discarded, the sedimented virions were resuspended in 250 μl PBS pH 7.2 containing 1 mM CaCl_2_ and the total protein concentration was determined using a BCA protein assay kit (Pierce) according to the 96-well plate protocol. All purified viruses were adjusted to a concentration of 1 mg/ml using PBS pH 7.2 containing 1 mM CaCl_2_, NA activity was measured using 0.5 μg and HAU titres were determined.

### NA activity measurements, IC_50_ determination and HAU titres

All NA activity measurements were performed in a 96-well low protein binding black clear bottom plate (Corning). Each sample was mixed with 37°C reaction buffer (0.1 M KH_2_PO_4_ pH 6.0 and 1 mM CaCl_2_) to a volume of 195 μl. Reactions were initiated by adding 5 μl of 2 mM MUNANA and the fluorescence was measured on Cytation 5 (Biotek) plate reader at 37°C for 10 min using 30 sec intervals and a 365 nm excitation wavelength and a 450 nm emission wavelength. Final activities were determined based on the slopes of the early linear region of the emission versus time graph. The zanamivir IC_50_ concentrations were determined similarly by including the indicated zanamivir concentrations in the reaction using 0.5 μg of purified virus. The slopes were then plotted against each zanamivir concentration and the IC_50_ value was determined using a variable slope four parameter nonlinear fit analysis in Graphpad Prism 8. HAU titres were determined by a two-fold serial dilution in a 96-well plate using a sample volume of 50 μl. Following the dilution, 50 μl of 0.5% TRBCs were added to each well and the plate was incubated 30 min at room temperature. HAU titres were determined as the last well where agglutination was observed.

### SDS-PAGE, Coomassie staining, immunoblotting, and densitometry

Purified virions equalling 5 μg of total viral protein (Coomassie), or 100 ng (immunoblots), were mixed with 2X sample buffer containing 0.1 M DTT as indicated. Samples were heated at 50°C for 5 min and resolved using a 4–12% polyacrylamide Tris-Glycine SDS-PAGE wedge gel. Gels were either stained with simple blue or transferred to a 0.45-μm pore PVDF membrane at 65 V for 1 h. PVDF membranes were blocked with milk/PBST (3% nonfat dry milk, PBS, pH 7.4, 0.1% Tween 20) for 30 min and processed with the indicated antibodies and appropriate HRP-linked secondary antibody. Immunoblots were developed with the SuperSignal West Femto kit (ThermoFischer) and imaged using an Azure C600 or a Syngene G Box. The same systems were used for Coomassie gel imaging. NA protein amounts in 5 μg of purified virions was determined by Coomassie gel densitometry analysis using purified recombinant N1-BR18 as a standard. Samples were resolved on the same 4–12% reducing SDS-PAGE gel, stained with simple blue, and imaged with an Azure C600. Densities of the NA bands were analysed using ImageJ and the protein amount was calculated using the standard curve from the recombinant protein samples.

### Bio-layer interferometry

Binding of the purified BPL inactivated virions to the 2,3-sialic acid analogue receptor, Neu5Ac-α2-3Gal-β1-4GlcNAc-β-PAA-biotin (3’SLN) was performed at 25°C using a ForteBio Octet Red96 bio-layer interferometer equipped with ForteBio Streptavidin biosensors at 25°C and a plate shake speed of 1000 rpm. Baselines were established for 2 min in 200 μl of buffer (PBS pH 7.4 containing 1mM CaCl_2_) prior to and post incubation of the biosensor with 200 μl of 6.7 nM 3’SLN for 10 min. Biosensors were then transferred to wells containing 200 μl of 0.1 mg/ml WSN^H1N1-BR18^ or PR8^H1N1-BR18^ virions in buffer with the indicated concentrations of zanamivir for 20 min. HA-mediated association was analysed by incubating the 3’SLN loaded biosensors in 200 μl of 0.1 mg/ml virions and 10 μM zanamivir for 20 min. NA-mediate dissociation was examined by transferring the biosensors to new wells that contained 200 μl of buffer for 20 min. Transfer to a well containing buffer with 10 μM zanamivir was included for a NA-inhibited dissociation control.

### EM analysis

Purified BPL inactivated virions in PBS pH 7.4, 1 mM CaCl_2_ with a total viral protein concentration of 1 mg/ml were diluted 1:4 in PBS pH 7.4 and 15 μl were spotted on formvar/carbon film 300 copper grids. Following a 5 min incubation, the virions were fixed with 2.5% Glutaraldehyde in 0.1 M PBS buffer pH 7.3 for 3 min, washed 3 times with dH_2_O, and stained with 2% Phosphotungstic Acid (PTA) for 50 sec. For each step the solution was drawn off the edge of the grid with filter paper. Grids were placed directly into a grid box and air-dried overnight before imaging with a Jeol 1400 transmission electron microscope (TEM) equipped with a Gatan US 1000XP digital camera.

### Immunizations, HAI and NAI titre measurements

Female DBA/2J mice were purchased from The Jackson Labs (#800671). At ~6 weeks of age, mice were immunized intramuscularly with the indicated protein amounts of purified BPL inactivated virions in 50 μl PBS. Mice receiving a second dose were immunized similarly 21 days after the first dose and blood was collected 21 days following the last immunization in Vacutainer SST II Advance Tubes (BD). The tubes were incubated at room temperature for ~1 h and placed at 4°C overnight. The next day the tubes were centrifuged (5 min; 2,000 x g) and the sera was transferred to a new tube and stored at -80°C for further analyses. A portion of the sera was treated with receptor destroying enzyme (RDE) overnight at 37°C and then heat inactivated at 56°C for 45 min. The HAI assay was performed in a 96-well plate using the PR8^H1N1-BR18^ virus in allantoic fluid. The virus was diluted to a titre of 4 HAUs and incubated with a two-fold serial dilution of the RDE treated sera in a total volume of 50 μl for 30 min at room temperature. Each well then received 50 μl of 0.5% TRBCs in PBS pH 7.2 and the plate was incubated for 45 min at room temperature. The HAI titre was calculated based on the sera dilution corresponding to the last well that prevented hemagglutination. NAI titres were determined using the enzyme-linked lectin assay (ELLA) with bovine fetuin and the PR8^H6/N1-BR18^ virus in allantoic fluid as described previously [62,63].

### Enzyme-linked immunosorbent assays (ELISAs)

HA ELISAs were performed by coating Immulon-2HB 96-well flat bottom plates with ~150 ng/well of rHA from A/Brisbane/02/2018 at 4°C overnight. Wells were blocked with 200 μl of PBS containing 1% BSA for 1 h at room temperature (RT) prior to the addition of the two-fold serially diluted mouse sera across the plate. Plates were incubated at 37°C for 1 h and the wells were three times with 200 μl/well of PBS containing 0.05% Tween. Goat anti-mouse IgG secondary was added for 1 h at RT, wells were washed and developed for 10 min at RT using 100 μL/well of OPD substrate dissolved in phosphate-citrate buffer. Reactions were stopped with 1N H_2_SO_4_ (50 μL/well) and read at 490 nm. HA endpoint titres were determined by the lowest sera dilution that gave an Abs_490nm_ greater than 0.1 or twice background. NA ELISA endpoint titres were determined similarly except Nickel coated plates were used to capture 250 ng/well of His-tagged rNA from A/Brisbane/02/2018 for 1 h at RT with shaking, wells were washed with a 20 mM Tris pH 7.5, 1 mM CaCl_2_, 150 mM NaCl, and 0.1% BSA buffer and the mouse sera were incubated overnight at RT.

### Statistical analysis

Data analysis was performed using GraphPad Prism 8 software under the assumptions that the data from the independent replicates follow a Gaussian distribution with equal standard deviation between samples. The *P* values were calculated either using Student’s unpaired t-test with a two-tailed analysis and a confidence interval (CI) of 95% or by a one-way ANOVA using Dunnett’s multiple comparisons test with a 95% CI. *P* values that were above 0.05 (not significant) were not included in any figures.

## Supporting information

S1 Data The raw measurement data for the graphs in each figure. (XLSX)
